# Hybrid Gradient Descent Grey Wolf Optimizer for Optimal Feature Selection

**DOI:** 10.1155/2021/2555622

**Published:** 2021-08-28

**Authors:** Peter Mule Kitonyi, Davies Rene Segera

**Affiliations:** Department of Electrical and Information Engineering, University of Nairobi, Nairobi 30197, Kenya

## Abstract

Feature selection is the process of decreasing the number of features in a dataset by removing redundant, irrelevant, and randomly class-corrected data features. By applying feature selection on large and highly dimensional datasets, the redundant features are removed, reducing the complexity of the data and reducing training time. The objective of this paper was to design an optimizer that combines the well-known metaheuristic population-based optimizer, the grey wolf algorithm, and the gradient descent algorithm and test it for applications in feature selection problems. The proposed algorithm was first compared against the original grey wolf algorithm in 23 continuous test functions. The proposed optimizer was altered for feature selection, and 3 binary implementations were developed with final implementation compared against the two implementations of the binary grey wolf optimizer and binary grey wolf particle swarm optimizer on 6 medical datasets from the UCI machine learning repository, on metrics such as accuracy, size of feature subsets, *F*-measure, accuracy, precision, and sensitivity. The proposed optimizer outperformed the three other optimizers in 3 of the 6 datasets in average metrics. The proposed optimizer showed promise in its capability to balance the two objectives in feature selection and could be further enhanced.

## 1. Introduction

With the advent of Big Data, more computation power and higher specialized methods are required to turn the collected statistics into valuable information and prediction strategies. Solutions to relate Big Data problems may be expressed as a function for which we seek to find an optimal solution, often with preset constraints. An analytic solution may end up as highly complex, requiring high computational power, or even nonexistent. In these common cases, an alternative method is required to efficiently comb through the search space of the problem to yield the optimal solution. Optimization techniques are well suited for this since they are search methods in which the objective is to find a solution for a given optimization problem.

Metaheuristic optimization algorithms, in specific, are viable owing to their simplicity, conceptuality, and analyticity. They guide the search process to explore the search space and arrive at an optimal solution [1]. Metaheuristic algorithms may be classified according to different characteristics, some of which include the following.

Memory usage vs. memoryless distinguishes whether the algorithm retains information of the already traversed search space and if this information is utilized in determining the algorithm's future actions. A well-known example of this is the swarm intelligence algorithm [2], which mimics the pheromone trails, produced by ants as they move to and from the colony, in encoding information of the search space.

Static and dynamic objective function metaheuristics refer to the morphing of the objective function as the search space is traversed as experienced in [3]; the algorithm dynamically modifies its objective function by analyzing the search space and adding constraints to the features that then alter the objective function through “penalty terms.”

Population-based vs. single point search optimizers describe the utilization of either a set of solutions referred to as search agents [4] or a single solution within the search space at each iteration [5], respectively.

One vs. various neighborhood structure metaheuristic algorithm is when a single search space is described by the objective function as opposed to multiple search spaces requiring the objective function to switch between different representations, as seen in [6].

Nature-inspired vs. non-nature-inspired simply specifies the insight that led to the creation of the algorithm. Many well-known metaheuristics are nature-inspired owing to the diverse and often ingenious methods through which nature has adapted to tackle naturally occurring optimization problems. The genetic algorithm, inspired by the process through which genetic information is encoded and handled in organisms to produce slight variations in offspring carrying traits of both parents, allows mutation and crossover in the parents' (the source of the genetic data) encoded features to produce offspring that may randomly acquire traits from either or both parents. From this idea, research was undertaken to create variants that exemplify the traits of the algorithm [7]. Some other algorithms are made after extensive research on natural processes in order to mimic nature such as the whale algorithm [8], dragonfly algorithm [9], and ant lion colony [10].

The grey wolf optimizer [11], inspired by the hunting patterns of the grey wolf (Canis lupus), is known for its well-defined pack leadership structure. The 4-tiered hierarchical hunting is encoded as tiered search agents, with the leaders driving the search direction and the rest encircling the prey (local minima).

Metaheuristic algorithms attempt to achieve a balance between diversification and intensification in the function space. As described in [12], an optimization algorithm cannot be a “one catch all,” having great performance values for all dataset types. This drives researchers to attempt to develop hybrid algorithms that incorporate the core of different algorithm ideologies to achieve a performance greater than the sum of its parts. This is especially critical in the medical field with numerous ailments requiring constant research to find relevant algorithms for each case.

With the simplification of data collection strategies, especially in internet-based processes, researchers have gained access to large repositories of data commonly referred to as Big Data. The data may be analyzed to understand the relations between different features or classify the set into distinct groups or predict future outcomes from it. Big Data tends to contain many features with some not affecting the classification of the data. The features are ignored, reducing computer workload and increasing accurate prediction of the algorithm in development.

The aim of feature selection is to filter out the features of a dataset that are redundant, not contributing to the prediction and labeling of the data [13], achieving data reduction. It helps in understanding a given data sample to know which of the input features significantly affect output. In industry, feature selection is vital in fault detection and diagnosis [14].

In the medical field, it may translate to a medical researcher gaining insight on specific metrics which are used to diagnose a specific ailment in a patient or create prediction algorithms for diagnosis. For diagnostic cases such as those used later in this paper, a researcher seeks to acquire as much information on a patient as possible. It is through feature selection that the researcher may input the data collected and gain insight on which of the features are relevant in the diagnosis of a patient, as in diagnosis and classification of neurodegenerative disorders [15]. Feature selection may also be used in preemptive testing of a patient and flagging a patient even before signs of illness are noticeable.

Feature selection's most common classification methods are as follows [14]: filter methods, embedded and hybrid methods, structured and streaming features, and wrapper (black box) methods, which are employed in this paper. The wrapper methods generate a subset of the features by the quality of the performance on the modeling algorithm, interacted with as a black box such that the internal mechanisms of operation of the evaluator are not manipulated during optimization; rather, the subsets are feed-in, and the output is generated as performance metric(s).

Stochastic gradient descent [16] is an optimization algorithm that utilizes partial differentials at the current solutions' spatial coordinates to traverse a search space and locate a local minimum. The algorithm effectively takes steps, at each iteration, in the direction away from the current gradient of the function. One of the main shortcomings of this algorithm is in its tendency to get trapped in a local minimum, unable to find the global minimum, especially in functions with an exceedingly high number of local minima, since the partial derivative also increases the computational complexity of the function by increasing the number of calculations required in iterating to the next point but in doing so informs the search agent on the most efficient direction to arrive at the minimum.

The aim of this paper is to propose a hybrid of the grey wolf and the gradient descent to achieve better performance in well-known medical benchmark datasets against established feature selection strategies by solving the feature selection problems.

The paper is subdivided into the following sections: introduction, literature review, review of the proposed method, results and discussion, and conclusion.

## 2. Literature Review

Binary GWO [17], proposed in 2015, modified the algorithm to operate in binary feature selection. This variant of the GWO operates on binary input variables as wolves denoting the selection of each feature. In this, a “1” in the vector includes the corresponding feature in the grey wolf's search space, while a “0” denotes an exclusion. Two approaches are proposed: (1) where the alpha, beta, and delta wolves are changed into binary vectors, after which a crossover is performed to find the value of the new grey wolf, and (2) where the GWO operates on the wolves as continuous-valued inputs to achieve a binary vector the wolves are subjected to a threshold or transfer function, specifically a sigmoid function. The optimizer's performance is compared against PSO [4] and genetic algorithms [7], with the outcome placing GWO as a viable candidate for feature selection problems.

In 2017, [18] was proposed. The novel framework is composed of the feature selection module and the KELM, evaluating the selected feature set. Compared against both the genetic algorithm and the GWO algorithm on multiple medical datasets, including Parkinson's and WDBC, the enhanced GWO achieves smaller feature subsets with higher performance metrics.

In 2017, a hybrid GWO was proposed by Singh and Singh [19]. It improved the exploitation of the base GWO by combining it with PSO. Compared against its parent optimizers, the hybrid was able to perform equivalently or markedly better in a set of benchmark equations yielding better solutions with comparatively reasonable CPU times [19].

Chaotic GWO [20], proposed in 2018, incorporates a set of chaotic maps to increase the convergence rate of GWO. Chaotic maps are used in optimization, taking advantage of their nature to generate highly variant randomness from “close” initial conditions. The modified GWO is applied to various design problems in engineering including the classic tension/compression spring design problem and the pressure vessel design problem, consistently outperforming algorithms such as the PSO and ant bee colony optimization algorithm [2].

In 2018, a binary grey wolf optimizer was used in feature selection for EMG signal classification in [21]. In the paper, the leaders are enhanced by integrating a random walk around the alpha, beta, and delta wolves to prevent trapping at the local optimum. The EMG signals, consisting of 120 features, are used to examine the effectiveness of the method with a KNN used for fitness evaluation. Compared against BGWO (both versions), we have BPSO and GA on classification accuracy, precision,*F*-measure, and MCC. The method reduces the features to an average of 42.46 and a precision of 0.9493, outperforming all the other algorithms on all metrics while averaging the lowest computation time.

In 2019, a method was presented to diagnose Parkinson's disease using modified grey wolf optimization [22]. The method involves removing the redundant features in the Parkinson's dataset using the modified GWO. With Parkinson's disease, early diagnosis is vital since, with no cure, treatment administered early helps with mitigation of its adverse effects. The Parkinson's datasets, split into training and testing and comprising hand, hand meander, speech, and voice, have many features on which the MGWO-driven feature selection, using a machine learning model as the source of the error rate, is run. In this paper, KNN, random forest, and decision tree performance were compared on the accuracy, detection rate, and false alarm rate on the 4 datasets with random tree performing better and thus used to compare against an optimized cuttlefish algorithm-based feature selection method. The proposed method achieved a higher accuracy in all datasets but a lower feature subset in 3 of the 4 datasets, proving the viability of the method and the applicability of GWO in feature selection.

In 2019, a modified binary grey wolf optimization method was proposed [23] to increase the accuracy of intrusion detection systems by applying feature selection to the data to select the optimal number of features. The modifications to the original grey wolf entailed having four wolves used in the position update instead of three and updating the fitness function to use the inverse number of selected features instead of the ratio of the number of selected features to the total number of features. The NSL-KDD dataset was used as the benchmark with a support vector machine for classification. The proposed algorithm was compared against variants of GWO on its average accuracy performance and average number of features with the algorithm achieving a higher average accuracy highlighting the impact of the two modifications applied to the GWO. The proposed method was then tested against BGWO, binary PSO, and binary BAT on an 8 to 2 train and test data split and 4 different attack methods, and the overall results showed no significant variance in best accuracy but a big difference in feature subset size. Convergence rate comparisons were also carried out against BGWO with the results indicating better evolution of the number of features with accuracy in the modified BGWO. The final simulation results showed an increase in the classification of 99.22% with a feature set reduction from 41 to 14. These results were compared against other state-of-the-art algorithms, including AdaBoost and PSO-discretize-HNB, indicating exceptional performance considering the conflicting objectives of the intrusion detection system. The authors proposed adapting a velocity parameter in future studies to enhance the performance as in PSO.

In 2019, in order to enhance the diagnosis of paraquat-poisoned patients, a herbicide commonly used for weeding, chaos enhanced grey wolf optimization wrapped ELM was proposed [24]. As with many ailments, early diagnosis increases the likelihood of recovery, and the proposed method was designed to remove redundant features from the dataset and enhance diagnosis accuracy. The chaotic sequence used in the paper was generated from logistic mapping and used to inject randomness and ergodicity and reduce sensitivity of the method to initial conditions. The grey wolf optimizer was used for feature selection to provide the optimal feature subset, with a decision boundary at 0.5 and an embedded chaotic map. An extreme learning machine model was trained and used to identify the PQ patients. The dataset used was from the Medical Ethics Committee of the First Affiliated Hospital of Wenzhou Medical University which included 15 patients. The results showed the method achieving AUC, accuracy, sensitivity, and specificity of 95.14%, 93.89%, 94.44%, and 95.83%, respectively, with the number of features selected ranging from 56 to 73 out of a total of 119 features. The significant features were indicated by the frequency of inclusion in the selected subset with feature nos. 3 and 87 being the most significant. The authors suggest orthogonal learning or quadratic interpolation to further enhance the searching of GWO.

In 2019, a face recognition method based on grey wolf optimization for feature selection [25] was proposed. In this paper, the authors used the grey wolf optimizer to prune out the redundant features in the image dataset and in doing so reduce the runtime of the process while increasing the classification accuracy. The dataset used in the paper was the Yale Face dataset which underwent image processing, feature extraction using Gabor filters, feature reduction using principal component analysis, feature selection using GWO, and classification using KNN. The effects of the GWO variables were analyzed for different values with accuracy peaking for the boundary of ±15 and maximum iterations of 25. The proposed system was compared against an adaptive cuckoo search algorithm for intrinsic discriminant analysis and outperformed it, achieving a higher accuracy (97% > 88.9%) with lower runtime (6.49 s < 9.10 s). This indicates the method is effective in face recognition and needs viable further research on other biometric datasets.

In 2020, improvements to the binary GWO were proposed [26] which involved a new updating equation for the “*a*” parameter to balance exploration and exploitation as well as different transfer equations for discretizing the output of the standard GWO positions. The transfer equations used are the standard *S* curve and 4 V-type functions which were further improved using the value of AD. To validate their application in feature selection, twelve datasets from the UCI machine learning repository were used on BGWO and the proposed algorithms. The KNN was used as the classification algorithm, and the fitness functions used were the *K*-fold loss and weighted sum ratio of *K*-fold loss and selected feature ratio. The simulated results prove that the improved optimizer has a better classification accuracy without increasing the selected features on a wide range of data types and dataset sizes. The authors suggested the use of a neural network for classification in combination with KNN to reduce classification error.

In 2021, a method was proposed [27] to tackle anomaly detection problems by utilizing an enhanced grey wolf optimizer in feature selection of the multidimensional dataset by controlling the balancing parameter for exploration and exploitation. The parameter “*a*” is the main focus with its value increased or decreased depending on the current iterations' performance as compared to the previous iteration. By doing so, the linearly updating value of “*a*” is changed to an adaptive update, allowing scouting of better search space when a wolf is at a worse fitness value area. The dataset used is the NSL-KDD dataset with 5 methods of attack with an SVM sued as the classifier. During phase one of analysis, the number of features and the accuracy were averaged and the proposed algorithm was compared against MBGWO, BGWO, MGWO, and GWO with the algorithm achieving the highest accuracy with the lowest number of features. The second phase pitted the algorithms on the accuracy and number of features selected for each class with the proposed algorithms' performance, indicating the advantage in parameter control with a good balance of exploration and exploitation. The results show significantly better performance of the optimizer, selecting 19 features of 41 with an 87.46% classification accuracy.

### 2.1. Inferences Drawn

The literature review shows the effectiveness of GWO and its variants in optimizations and feature selection in specific, with GWO simplicity in design and implementation, few controlling parameters, and ease of modifying with other optimizers. It also shows that the wrapper method is a superior feature selection method with the KNN classifier acting as a suitable error rate generator without leading to overfitting.

## 3. Grey Wolf Optimizer

Proposed by Mirjalili et al. in 2014 [11], the grey wolf optimizer is a static population-based metaheuristic optimization algorithm inspired by the grey wolf of the Canidae family. The algorithm mimics the search, hunt, and attack tactics of a pack of grey wolves as a cohesive unit.

**Figure 1:**
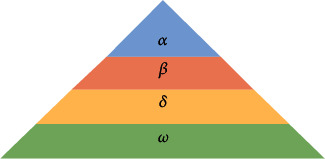
Hierarchy of the wolves in GWO.

Within a pack of wolves, there exists a strict social hierarchy divided into four tiers depicted in Figure 1.

*(i) The Alpha (α*). The leader of the pack is responsible for hunting, scheduling among other decisions concerning the pack.

*(ii) The Beta (β*). Subordinates to the alphas assist them in the management and decision-making of the pack; they are usually the first in line to acquire the title of alpha if the current alpha passes on or grows too old. They also act as an enforcer to the alpha on the rest of the pack, disciplining them as need be.

*(iii) The Delta (δ*). Dominant only to the omegas, this group is mostly composed of the sentinels, elders, hunters, scouts, and caretakers.

*(iv) The Omega (ω*). Often referred to as the scapegoat, these wolves are inferior to all the other wolves seeking guidance from all other wolves.

The pack leaders are the spearheads in a hunting formation. They send the omega wolves to encircle the prey once its general location is found, drawing closer and closer as the exact location is searched for. With the prey completely encircled, the wolves attack, securing a meal for the pack.

To express this as a mathematical model, the process may be divided into 3 distinct steps.

### 3.1. Encircling the Prey

(1) $$\begin{array}{c} \overset{⟶}{D}=\left|\overset{⟶}{C}\cdot {\overset{⟶}{X}}_{\text{p}}\left(t\right)-\overset{⟶}{X}\left(t\right)\right|, \end{array}$$

(2) $$\begin{array}{c} \overset{⟶}{X}\left(t+1\right)={\overset{⟶}{X}}_{\text{p}}\left(t\right)-\overset{⟶}{A}\cdot \overset{⟶}{D}. \end{array}$$

The encircling of the prey is modeled as in equation (1).

$\overset{⟶}{D}$ is calculated as the distance between the current wolf vector $\overset{⟶}{X}\left(t\right)$ and the prey ${\overset{⟶}{X}}_{\text{p}}$.

$\overset{⟶}{X}\left(t+1\right)$ is the next value of *X*, and $\overset{⟶}{A}$ and $\overset{⟶}{C}$ are random vectors of dimensions equal to the dimensions of *X* generated from ${\overset{⟶}{r}}_{1}$ and $\overset{⟶}{r_{2}}$ of the range [0, 1] and with a range of [0, 2].

(3) $$\begin{array}{c} \overset{⟶}{A}=2\overset{⟶}{a}\cdot {\overset{⟶}{r}}_{1}-\overset{⟶}{a}, \end{array}$$

(4) $$\begin{array}{c} \overset{⟶}{C}=2\cdot \overset{⟶}{r_{2}}. \end{array}$$

$\overset{⟶}{a}$decreases from 2 to 0 that models the circling of the prey, as the iteration counts up, as expressed in equation ((5)) below:

(5) $$\begin{array}{c} \overset{⟶}{a}=2-\left(\frac{2\times \text{ⅈtⅇr}}{\text{Ma}{\text{x}}_{\text{iter}}}\right). \end{array}$$

### 3.2. Hunting the Prey

Hunting maps explore the search space as led by the alpha, beta, and delta. Modeling this involves obtaining the alpha, beta, and delta search agents from the pack by comparing the fitness values and choosing the leading agents. The omega positions are then updated according to the leading wolves.

(6a) $$\begin{array}{c} \overset{⟶}{D_{\alpha }}=\left|\overset{⟶}{C_{1}}\cdot {\overset{⟶}{X}}_{\alpha }-\overset{⟶}{X}\right|, \end{array}$$

(6b) $$\begin{array}{c} \overset{⟶}{D_{\beta }}=\left|\overset{⟶}{C_{2}}\cdot {\overset{⟶}{X}}_{\beta }-\overset{⟶}{X}\right|, \end{array}$$

(6c) $$\begin{array}{c} \overset{⟶}{D_{\delta }}=\left|\overset{⟶}{C_{3}}\cdot {\overset{⟶}{X}}_{\delta }-\overset{⟶}{X}\right|, \end{array}$$

(7a) $$\begin{array}{c} {\overset{⟶}{X}}_{1}={\overset{⟶}{X}}_{\alpha }-{\overset{⟶}{A}}_{1}\cdot \left({\overset{⟶}{D}}_{\alpha }\right), \end{array}$$

(7b) $$\begin{array}{c} {\overset{⟶}{X}}_{2}={\overset{⟶}{X}}_{\beta }-{\overset{⟶}{A}}_{2}\cdot \left({\overset{⟶}{D}}_{\beta }\right), \end{array}$$

(7c) $$\begin{array}{c} {\overset{⟶}{X}}_{3}={\overset{⟶}{X}}_{\delta }-{\overset{⟶}{A}}_{3}\cdot \left({\overset{⟶}{D}}_{\alpha }\right), \end{array}$$

(8) $$\begin{array}{c} \overset{⟶}{X}\left(t+1\right)=\frac{{\overset{⟶}{X}}_{1}+{\overset{⟶}{X}}_{2}+{\overset{⟶}{X}}_{3}}{3}. \end{array}$$

### 3.3. Searching and Attacking the Prey

Once the prey stops moving, the pack moves in for the kill, sending all the agents towards the prey from different angles.

The mathematical model of these is dependent on vector $\overset{⟶}{A}$. When the absolute value of vector $\overset{⟶}{A}$ is below 1, the search agents converge towards the prey when it is above the agents that search the space.

## 4. Iterative Stochastic Gradient Descent

Conceptualizing an objective function as an unknown terrain, the minimum of this terrain may be approached step by step, continually moving against the gradient of the terrain.

Gradient descent implements this as an iterative update process of the position *θ*.

(9) $$\begin{array}{c} \theta =\theta -\eta \nabla_{\theta }J\left(\theta ;x^{\left(i\right)};y^{\left(i\right)}\right). \end{array}$$

For a function whose objective function is defined as *J*(*θ*; *x*^(*i*)^; *y*^(*i*)^), whose partial derivative with respect to each parameter of*x*^(*i*)^is∇_*θ*_*J*(*θ*; *x*^(*i*)^; *y*^(*i*)^), the core equation of gradient descent is shown in equation (9).

The algorithm loops a set number of times (maximum iterations); at each iteration, the value of *θ* is updated by calculating the partial derivative of the objective function with respect to the parameters of the input and subtracting this value from *θ*. In doing so, the algorithm quantifies the effect each parameter has on the objective function and uses this information to control the direction and speed of transversal in the search space. The variable *η* controls the learning rate, avoiding both oscillations about a minimum caused by large values of the partial derivative and slow convergence rates caused by low partial derivative values.

For a simple function space, the partial derivative encodes the direction in which the closest local minimum exists. As the function increases in complexity with multiple local minima existing, the value of the partial derivative as a vector in the function space may point at the weighted average of the local minima. To avoid this outcome, the algorithm is usually run multiple times with each initial starting location randomized.

This algorithm is well known in implementation in artificial intelligence, specifically in regression and neural networks. With a mathematically derived partial derivative, the algorithm can achieve a high convergence rate, and with multiple random initializations, it avoids local minima.

One of its significant shortcomings is that when the partial derivative calculation is intensive, the computational complexity of the overall process is increased.

## 5. Feature Selection

Feature selection is the process of decreasing the number of features in a dataset by removing redundant, irrelevant, and randomly class-corrected data features. In doing so, a model is capable of increasing its accuracy as well as reducing overfitting and training time by utilizing the generated optimal subset. It has applications in many fields including text mining, image processing, medical research, and fault diagnosis.

**Figure 2:**
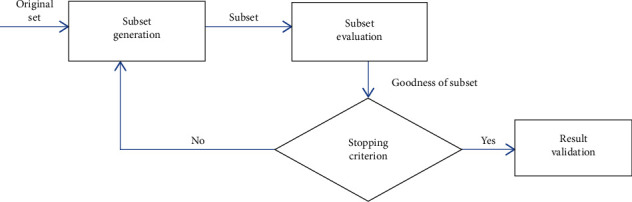
Feature selection process.

The general procedure of feature selection involves four key steps [28], as shown in Figure 2:

*(i) Subset Generation*. This is the start of the process as a heuristic search. It could be a forward search which involves starting with an empty feature set and successively adding new features or a backward search which involves starting with all features included and successively removing features or a bidirectional search which involves adding and removing features simultaneously.

*(ii) Subset Evaluation*. With a generated feature subset, this involves determining the “goodness” of the subset through a defined criterion through which the optimal subset of features is guided. This may be dependent or independent of the mining algorithm.

*(iii) Stopping Criterion*. This determines when the feature selection process terminates through criteria such as search completion, reaching of specified bounds (minimum no. of features or maximum number of iterations), an optimal feature subset obtained with alteration of the subset not providing better subsets, and a subset selected with sufficient criterion values.

*(iv) Result Validation*. The selected feature subset is selected, and its performance is evaluated.

The methods of feature selection may be classified into the following [14]:

*(1) Filter Methods*. Features are selected based on performance metrics, and the dataset used is not taken into account.

*(2) Wrapper Methods*. The performance of a given feature subset is measured by a modeling algorithm whose internal workings are “not known.” Owing to this, they are versatile, allowing any pairing of the modeling algorithm and search space optimization algorithm. This is done for each iterative subset as per the algorithm in use until the stopping criterion is met. These methods are slower than filters but provide better performance given that the subsets are ensured not to be biased to the modeling algorithm used.

*(3) Embedded and Hybrid Methods*. Feature selection occurs during the modeling algorithm execution. As a model is being generated from the test data, the redundant features are pruned out to achieve the optimal dataset and model at completion. This is a hybrid of both the wrapper and filter methods.

## 6. Proposed Method

### 6.1. Hybrid Gradient Descent Grey Wolf Optimizer

The gradient descent of a wolf position may be conceptualized as the direction from which the wolf seems to smell the prey is at currently. The leading wolves would then smell the air, and each chooses a representative among the worst-performing members of the pack and calls them to itself, then instructs it to “follow” the scent. The basic implementation is given in Pseudocode 2 below.

The partial derivative is calculated as an approximation since the objective function is taken as unknown. This is done by choosing a step variable that is the value added to the wolf position on each dimension as well as subtracted. The fitness values of these two new positions are calculated and subtracted from each other and lastly divided by the step value. This is the center finite difference for derivative approximation.

(10) $$\begin{array}{c} \frac{\partial J\left(\overset{⟶}{x}\right)}{\partial x_{i}}=\frac{J\left(x_{1},x_{2},\cdots ,\left(x_{i}+s\right),\cdots ,x_{n}\right)-J\left(x_{1},x_{2},\cdots ,\left(x_{i}-s\right),\cdots ,x_{n}\right)}{s}. \end{array}$$

### 6.2. HGDGWO (Binary Version)

The binary version adapts the proposed continuous algorithm for feature selection while taking advantage of the finite search space of a dataset: each location in space either including or excluding a feature from consideration in the model.

As a multiobjective feature selection problem, the fitness function adapts to this as a weighted sum of the two opposing solution requirements, fewer selected features and low error rates.

A significant alteration is in the calculation of the fitness function. Since there are two objectives of the fitness function, in order to incentivize the algorithm to seek out solutions with fewer features, a “cost” is assigned to the number of features and added to the fitness function. It is calculated as below:

(11) $$\begin{array}{c} \text{fit}=\phi \text{Err}+\theta \frac{S}{D}, \end{array}$$

where *ϕ* modulates the error of the fitted model, Err, and *θ* = (1 − *ϕ*) modulates the ratio of the number of selected features *S* to the total number of features *D*.

The alterations to the partial derivative for a binary search space are shown in Pseudocode 3 below. For the partial derivative, each feature index goes through the binary NOT operation, and this value is subtracted from the original partial differential.

Once the partial derivative is calculated, this information is utilized in updating the wolf positions through the mutation by partial derivative functions. Three implementations were used as follows:

1. With this implementation, shown in Pseudocode 4, the partial derivative is used as a simple threshold marker with higher derivatives leading to lower feature index inversion rates. The higher the partial derivative, the higher the chance of changing the wolves' value at the partial derivative index
2. In this implementation, shown in Pseudocode 5, variable *a* is used to incorporate exploration and exploitation phases during the iterative search process. It does this by modifying the threshold for the wolf index mutation
3. With this, shown in Pseudocode 6, the sigmoid function is used to map the partial derivative to a threshold space with a normalized partial derivative while still incorporating variable *a* for exploration and exploitation. This ensures injection of inversion even when there seems to be no valid update direction for the wolf

With mutation completed, the worst-performing wolves' positions are updated as the modified positions are evaluated. The three worst-performing wolves were chosen to update the positions from the alpha, beta, and delta wolves.

## 7. Methodology

The algorithms were implemented in MATLAB and run on an Intel® Core™ i7-7700HQ CPU @ 2.80 GHz with 8 GB of RAM.

The continuous version was first tested for feasibility and gave information on its performance in different continuous function optimization problems. The binary version underwent multiple alterations to improve performance.

### 7.1. HGDGWO

As a continuous value optimization function, the hybrid algorithm was tested on 23 benchmark functions. Each function was run 500 times, and the convergence curve and final value were recorded.

#### 7.1.1. Test Functions

**Figure 3:**
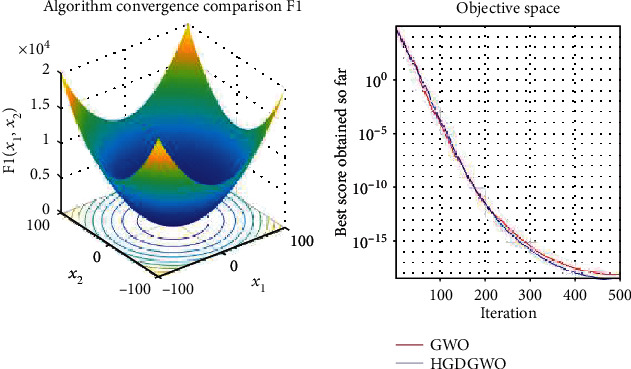
Convergence graph of unimodal benchmark function (F1). GWO indicates grey wolf optimization; HGDGWO indicates hybrid gradient descent grey wolf optimizer.

**Figure 4:**
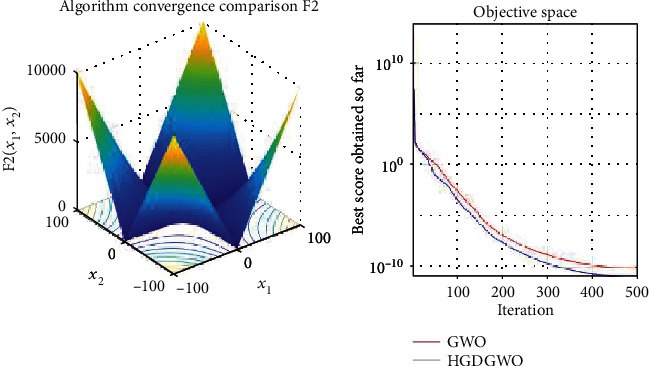
Convergence graph of unimodal benchmark function (F2). GWO indicates grey wolf optimization; HGDGWO indicates hybrid gradient descent grey wolf optimizer.

**Figure 5:**
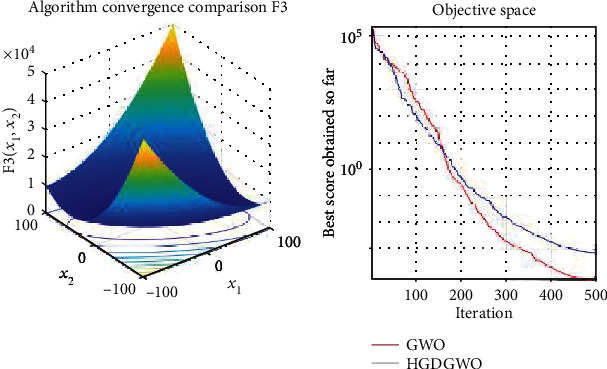
Convergence graph of unimodal benchmark function (F3). GWO indicates grey wolf optimization; HGDGWO indicates hybrid gradient descent grey wolf optimizer.

**Figure 6:**
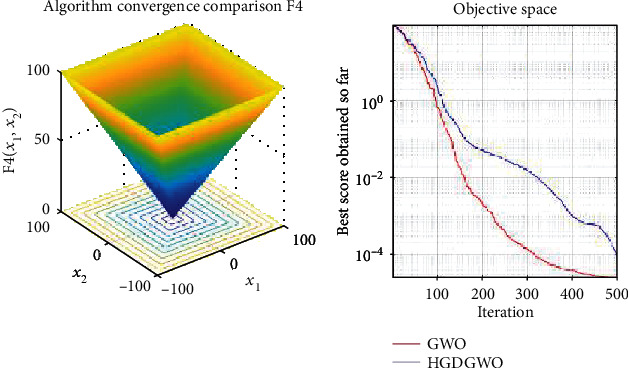
Convergence graph of unimodal benchmark function (F4). GWO indicates grey wolf optimization; HGDGWO indicates hybrid gradient descent grey wolf optimizer.

**Figure 7:**
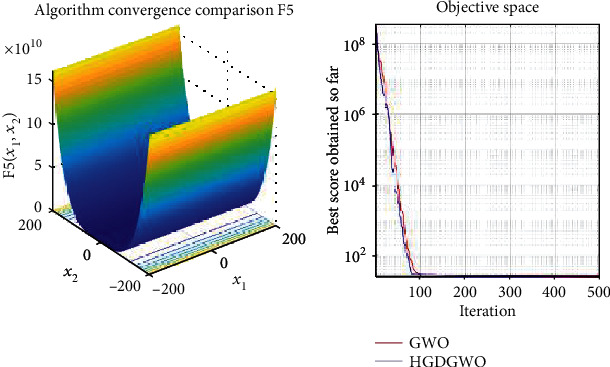
Convergence graph of unimodal benchmark function (F5). GWO indicates grey wolf optimization; HGDGWO indicates hybrid gradient descent grey wolf optimizer.

**Figure 8:**
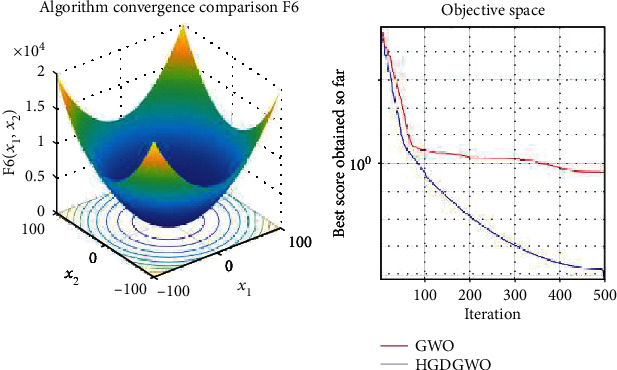
Convergence graph of unimodal benchmark function (F6). GWO indicates grey wolf optimization; HGDGWO indicates hybrid gradient descent grey wolf optimizer.

**Figure 9:**
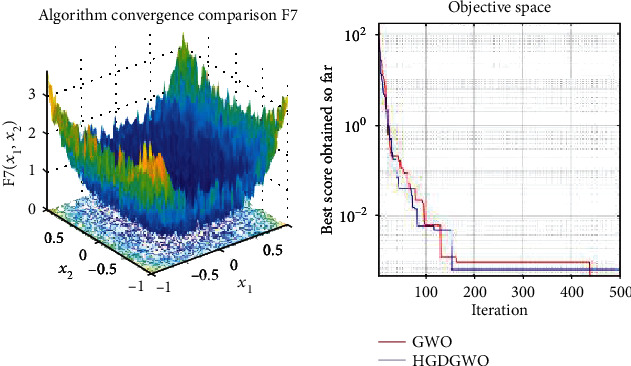
Convergence graph of unimodal benchmark function (F7). GWO indicates grey wolf optimization; HGDGWO indicates hybrid gradient descent grey wolf optimizer.

Twenty-three benchmark functions were used to compare the performance of the HGDGWO against GWO. They are divided into three types of functions:

*(1) Unimodal Functions (F1-F7)*. Exploitation analysis for checking the exploitation capability of the optimizer (Figures 3–9).

**Figure 10:**
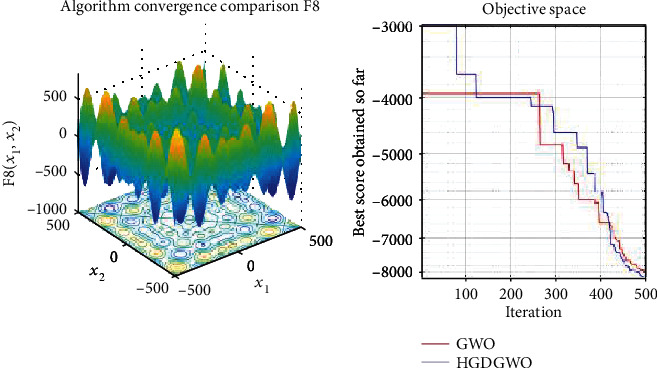
Convergence graph of multimodal benchmark function (F8). GWO indicates grey wolf optimization; HGDGWO indicates hybrid gradient descent grey wolf optimizer.

**Figure 11:**
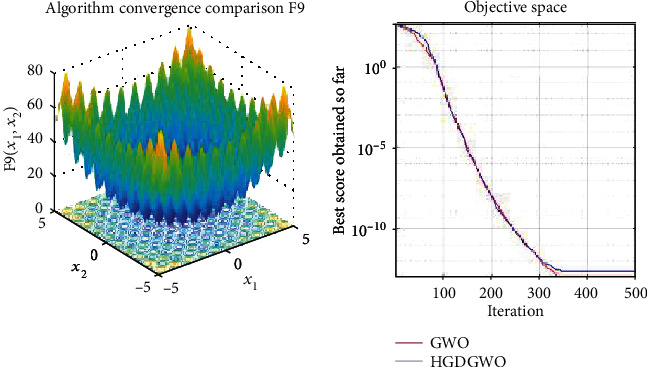
Convergence graph of multimodal benchmark function (F9). GWO indicates grey wolf optimization; HGDGWO indicates hybrid gradient descent grey wolf optimizer.

**Figure 12:**
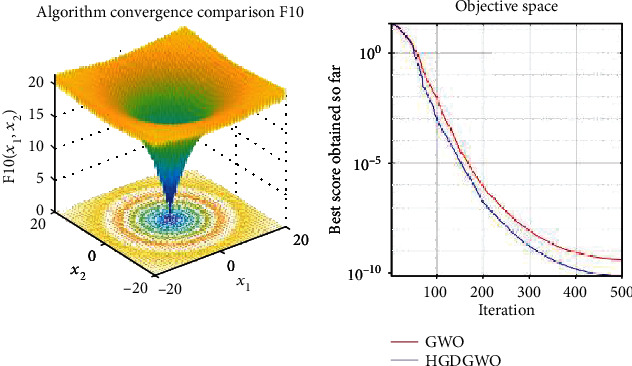
Convergence graph of multimodal benchmark function (F10). GWO indicates grey wolf optimization; HGDGWO indicates hybrid gradient descent grey wolf optimizer.

**Figure 13:**
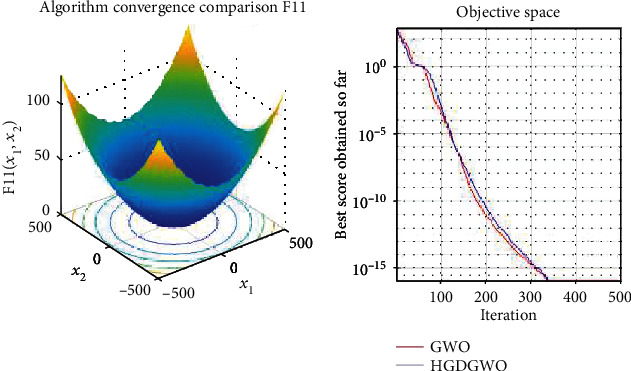
Convergence graph of multimodal benchmark function (F11). GWO indicates grey wolf optimization; HGDGWO indicates hybrid gradient descent grey wolf optimizer.

**Figure 14:**
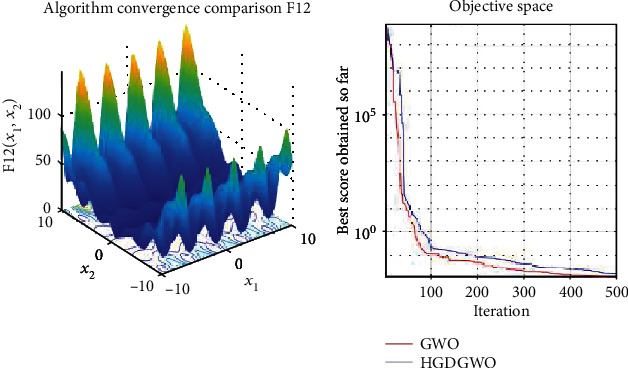
Convergence graph of multimodal benchmark function (F12). GWO indicates grey wolf optimization; HGDGWO indicates hybrid gradient descent grey wolf optimizer.

**Figure 15:**
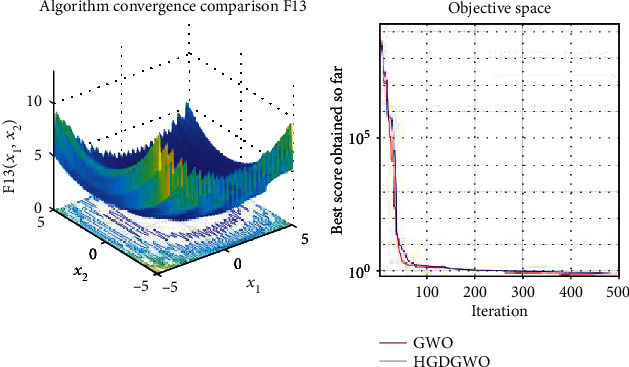
Convergence graph of multimodal benchmark function (F13). GWO indicates grey wolf optimization; HGDGWO indicates hybrid gradient descent grey wolf optimizer.

*(2) Multimodal Functions (F8-F13)*. Exploration analysis for checking the exploration capability of the optimizer (Figures 10–15).

*(3) Fixed-Dimension Multimodal Functions (F14-F23)*. For analysis of the exploration capability of the algorithm in the case of fixed-dimension optimization problems (Figures 16–25).

#### 7.1.2. Parameter Settings

- Number of test cases = 500
- Maximum iterations = 500
- Number of wolves = 10
- Learning rate *η* = 0.003. This is a variable for the partial derivative, defining the position update rate. A high learning rate increases the convergence rate while increasing oscillations around a local minimum
- Step size *s* = 1 *E*‐15. For partial derivative approximation, a high value reduces the accuracy of the value calculated

The parameters were tuned iteratively in order to achieve desired results.

#### 7.1.3. Evaluation Metrics

1. Standard deviation

(12) $$\begin{array}{c} \sigma^{j}=\sqrt{\frac{\sum_{i=0}^{N}{\left(S_{i}^{j}-\mu \right)}^{2}}{N}}. \end{array}$$

This is a measure of the similarity between different solution runs. A high standard deviation indicates significant changes in the solution as the function runs multiple times. A low variance indicates a relatively static solution irrespective of the number of reinitializations.

(2) Average of solutions

(13) $$\begin{array}{c} \mu^{j}=\frac{\sum_{i=0}^{N}S_{i}^{j}}{N}. \end{array}$$

(3) Minimum solution

(14) $$\begin{array}{c} \text{Minimum}=\min\left(S^{j}\right). \end{array}$$

This is the lowest value of the fitness value achieved over the total number of repetitions.

(4) Timing

The MATLAB timing function “timeit” is used. The algorithms are timed on how long they take on each function, from calling to returning the solution.

### 7.2. HGDGWO (Binary Version)

#### 7.2.1. Datasets

From the UCI machine learning repository, 6 medical datasets were selected and utilized to test HGDGWO in feature selection applications against BGWO implementations 1 and 2 as well as BGWOPSO.  Table 1 shows the specific datasets used and the corresponding feature numbers and samples.

*(1) Breast Cancer Wisconsin (Diagnostic)*. Features are computed from a digitized image of a fine needle aspirate (FNA) of a breast mass. They describe the characteristics of the cell nuclei present in the image.

*(2) Breast Cancer Wisconsin (Original)*. Some of its features are clump thickness, uniformity of cell shape, uniformity of cell size, and single epithelial cell size, among others.

*(3) SPECT Heart*. The dataset describes diagnosing cardiac Single Photon Emission Computed Tomography (SPECT) images. Each of the patients is classified into two categories: normal and abnormal.

*(4) Statlog (Heart)*. The class is grouped as either the absence (1) or the presence (2) of heart disease. The data includes patient information and symptoms as well as medical test results. It includes features such as age, sex, chest pain type, resting blood pressure, cholesterol, and fasting blood sugar.

*(5) Lymphography*. The dataset is grouped into 4 classes: normal find, metastases, malign lymph, and fibrosis. The features are characteristics of the nodes of the patient including shape, defects, and extravasates. Some of the classes are disproportionally represented, influencing the partitioning of the dataset.

*(6) Cleveland Heart Disease (Coronary Artery Disease)*. The dataset includes patient information such as age, sex, fasting blood sugar, and cholesterol, mapping to the patient diagnosis.

#### 7.2.2. Parameter Settings

- Number of wolves = 10
- Maximum number of iterations = 50
- Number of tests = 10
- Random wolf initialization threshold = 0.3. This variable sets the number of initial selected features with higher values translating to lower initial selected features
- Partial derivative mutation threshold = 0.9. This operates as a shift in threshold position for altering the chance of inversion of a feature
- Limits of exploitation vs. exploration weight = [0.1,0.9]. This sets the limits for the probability of exploration and exploitation

The parameters were tuned iteratively in order to achieve desired results.

#### 7.2.3. Feature Selection

The feature selection method used was the wrapper-based method with the following presets:

(i) Objective function is the partial derivative, as expressed in equation (10)
(ii) Search strategy is HGDGWO (random bidirectional)
(iii) Modeling algorithm is KNN with a *K* value = square root of the test cases
(iv) Distance calculation function is the Euclidean distance
(v) *K*-fold is 5
(vi) External classifier is the support vector machine
  - Standardize is true
  - Kernel function is RBF
  - Kernel scale is auto
(vii) Data splitting function cv-partition and number of partitions = 10 for 2 class datasets and 2 for classes more than 2 with the distribution skewed

The wrapper method was chosen for its superior performance, as expressed in the literature review. The KNN classifier was also used for fitness evaluation because of its well-documented preference in feature selection, specifically with GWO variants.

#### 7.2.4. Evaluation Metrics

The criteria used once a solution was achieved were as follows:

1. Average classification accuracy

This is a measure of the validity of a model's predictions.

(15) $$\begin{array}{c} \text{Average accuracy}=\frac{1}{K}\overset{K}{\underset{i=1}{\sum }}{\text{Acc}}^{i}. \end{array}$$

(2) Average number of selected features
(3) Average fitness values
(4) Sensitivity

This is the ratio of correctly predicted positive cases to total positive cases.

(16) $$\begin{array}{c} \text{Sensitivity}=\frac{\text{TP}}{\text{TP}+\text{FN}}. \end{array}$$

(5) Precision

This is the ratio of correctively predicted positive cases to total predicted positive cases.

(17) $$\begin{array}{c} \text{Precision}=\frac{\text{TP}}{\text{TP}+\text{FP}}. \end{array}$$

(6) *F*-measure

This is the harmonic average of precision and sensitivity.

(18) $$\begin{array}{c} F‐\text{measure}=2\frac{\text{Precision}*\text{Recall}}{\text{Precision}+\text{Recall}}. \end{array}$$

(7) One-way ANOVA test on the number of features selected by each algorithm. The *P* value was obtained from the MATLAB function “anova1”

## 8. Results and Discussion

### 8.1. HGDGWO Results

The proposed HGDGWO is compared against GWO and manages to achieve higher average fitness values for 11 of the 23 fitness functions but achieves a lower minimum fitness value in 14 of the 23, as seen from Table 2.

The optimizer also increases the average computation time by a factor of 5.6, as seen from Table 3.

From the graphs, the proposed function achieves a higher convergence rate for functions 2, 6, 10, 14, 15, 20, 21, 22, and 23, as seen in Figures 4, 8, 12, 16, 17, 22, 23, 24, and 25.

In only functions 3, 4, and 12, in Figures 5, 6, and 14, does the hybrid optimizer perform noticeably worse than the original GWO with each solution starting out at the same rate but diverging as the function traverses the search space.

With functions 1, 5, 7, 8, 9, 11, 13, 16, 17, 18, and 19 from Figures 3, 7, 9, 10, 11, 13, 15, 18, 19, 20, and 21, the convergence curve closely matches the GWO curve with an almost equal rate. This range includes almost all the multimodal functions, indicating that the hybrid function does not increase the optimizer's convergence rate in multimodal functions and even reduces its performance, as in the case of function 12 (Figure 14).

Performance of the HGDGWO among F1-F7 indicates superiority in exploiting the optimum. The performance in multimodal functions is also promising, indicating that the optimizer would also perform well in objective functions with a large number of local minima.

This indicated the virility of this hybrid function as a possible solution to the optimization function.

The optimizer also increases the average computation time by a factor of 5.6, as seen from Table 3. This was as expected since the partial derivative required in calculation increases the computation time.

### 8.2. HGDGWO (Binary Version) Results

#### 8.2.1. Implementation 1

Table 4 shows the performance of the HGDGWO against the BGWO on the datasets stated in Table 1.

With this implementation, HGDGWO performs markedly better than BGWO2 in accuracy, *F*-measure, precision, and sensitivity, outperforming it in all datasets apart from SPECT Heart. Although the results were promising, the number of features selected was higher for the markedly low increase in performance, indicating the persistence of redundant features in the feature subsets produced by HGDGWO.

#### 8.2.2. Implementation 2

The results presented below indicate the performance of HGDGWO's second implementation against the two implementations of the binary GWO. The results are from 50 iterations of 10 test cases each and tabulated as average maximum and minimum values for the 4 datasets.

As seen from Table 5, HGDGWO performs averagely for all metrics apart from sensitivity, performing better than the BGWO2 but worse than BGWO1 on average, but has similar performance in maximum values. In both *F*-measure and precision, the difference is marginal (~0.01). The maximum metric values for the resultant predictor were identical, each optimizer managing to achieve unity.

As seen from Table 6, HGDGWO outperforms the other optimizers in accuracy, *F*-measure, and precision of the predictor. The number of maximum features selected for both HGDGWO and BGWO2 is 3 out of the possible 9 with none exceeding 3 features apart from BGWO1.

From Table 7, HGDGWO outperforms the BGWO in all predictor metrics but performs medially in both the fitness values and the number of features in the average values. The function also has the best performance in the maximum values.

In this dataset, it is noted that the difference between the maximum and minimum metrics for all three optimizers is very large, indicating a need to run them multiple times to achieve a satisfactory value. This is an effect of a local minimum, trapping the optimizer in some of its iterations.

The results from Table 8 show that BGWO2 outperforms the other two functions in the average values, but in the maximum values, HGDGWO has the best performance in accuracy, precision, and *F*-measure; this indicates that although averagely HGDGWO performance is low, when run multiple times, it achieves a higher performing predictor from the selected feature set.

#### 8.2.3. Implementation 3

The results presented below indicate the performance of HGDGWO's third and final implementation against the two implementations of the binary GWO as well as the binary GWO and PSO hybrid. The results are from 50 iterations of 10 test cases each for the 5 datasets and 2 test cases for the Lymphography dataset.

The evaluation is done on the average, minimum, and maximum values of each criterion, using the one-way ANOVA*P*test on the number of features selected with the box plot and comparison of criteria for the feature subset with the highest accuracy.

*(1) Maximum, Minimum, and Average Value Comparison*. As seen from Table 9, the proposed optimization function does not outperform the benchmark functions but achieves the second smallest average feature subset. With a higher *F*-measure than the smallest average feature subset as from BGWO2, the proposed algorithm shows a better balance in the error rate and feature subset size, especially since the higher performing algorithm also has the largest feature subset average.

The proposed algorithm also achieves the highest possible maximum values in accuracy, *F*-measure, precision, and sensitivity with 9 maximum features selected which BGWO2 is unable to.

In this dataset, from Table 10, the algorithm averages the lowest number of features with a difference of 3.3 from the maximum in the selected features. With a lower feature subset, it achieves an accuracy difference from BGWOPSO of 0.03. There is a low deviation in the number of features with the maximum at 3 and minimum at 2 from a possible maximum of 9, the same as in HGDGWO2.

The maximum values also indicate that with a smaller average feature subset by 5, the proposed method still manages to achieve unity in the classification parameters.

As in HGDGWO2, from Table 11, implementation 3 outperforms all other functions as well as outperforming HGDGWO2 in accuracy, *F*-measure, fitness values, and sensitivity, with an averagely lower number of features than HGDGWO2.

The difference in maximum and minimum values of the classification parameters of the proposed method is large, indicating that the method may be getting trapped in local minima.

As seen from Table 12, the proposed function is only outperformed by the BGWO2 function. The average accuracy difference is ~0.2 for a 2.58 average feature subset reduction.

In this subset, as seen from Table 13, the proposed algorithm averages a higher value of *F*-measure and sensitivity with an average of 4.6 features while BGWOPSO averages a higher accuracy and precision with an average of 4 features.

With this dataset, as seen from Table 14, the accuracy plummets to exceedingly low values, <0.59 for all algorithms. The proposed algorithm achieves the highest average *F*-measure but with the largest average feature subset.

From the results (Tables 9–13), the optimizer manages to attain a balance between the performance and the fitness values as set by the value *ϕ* from equation (10). The variable is set not to overemphasize the significance of one over the other. This is especially critical in the stopping criterion stage of the feature selection criterion. When the limiting criterion is set as the number of selected features, the value of the variable is reset to convey this. HGDGWO2 performs better in the breast cancer dataset while HGDGWO performs better with the heart dataset.

*(2) Highest Accuracy Feature Subset Performance Comparison*. As seen from Table 15, the proposed method achieves unity classification metrics with a 7-feature subset which is the least with all unity. Inversely, BGWO2 has a lower feature subset but in doing so does not achieve unity classification.

From Table 16, HGDGWO achieves unity on accuracy, *F*-measure, precision, and sensitivity with the smallest feature subset, selecting 2 features as relevant out of the possible 9.

From Table 17, the proposed function seems to have been trapped in a local minimum, having selected the lesser performing set of 4 features, but achieves an equivalent value of accuracy, *F*-measure, precision, and sensitivity with BGWO1 with fewer features.

In Table 18, the accuracy caps out at 0.8889. With 2 features resulting in the highest metrics, the proposed method was unable to achieve this, instead having an 8-feature subset, the second best.

In Table 19, the proposed method achieves the highest sensitivity and accuracy with 5 features and with the second *F*-measure accuracy, a difference of 0.02, compared to a possible 13-feature subset. It achieves a good balance of the two objectives.

In Table 20, though the HGDGWO feature subset is among the smallest, the performance of the algorithm in this subset is still subpar, indicating that the search objective was hampered, possibly by the local minima.

#### 8.2.4. One-Way ANOVA Test on Feature Data

The stated hypotheses of the one-way ANOVA test on the number of features are as follows:

*(i) H0*. The null hypothesis indicates that there is no significant difference between the number of features of HGDGWO and the other methods used in testing.

*(ii) H1*. The alternative hypothesis indicates that there is a significant difference between the number of features of HGDGWO and the other methods used in testing.

The significance of the ANOVA test results is obtained from comparison against the standard significance level of 0.05 with *P* values greater than the stated value, indicating acceptance of the null hypothesis and rejection of the alternative hypothesis, and with *P* values less than the stated value, indicating rejection of the null hypothesis and acceptance of the alternative hypothesis.

From the*P*value results in Table 21, the Breast Cancer Wisconsin (Original) and Lymphography datasets' BGWO2 vs. HGDGWO3 and the heart disease dataset's BGWOPSO vs. HGDGWO3 show that the*P*test values are not lower than the significance level of 0.05 which leads to the rejection of the alternative hypothesis and acceptance of the null hypothesis.

The other comparative *P* values indicate a rejection of the null hypothesis of no significant difference between the number of features. The general *P* value comparing all data points of all the algorithms shows that there is a significant difference between at least one of the samples.

**Figure 26:**
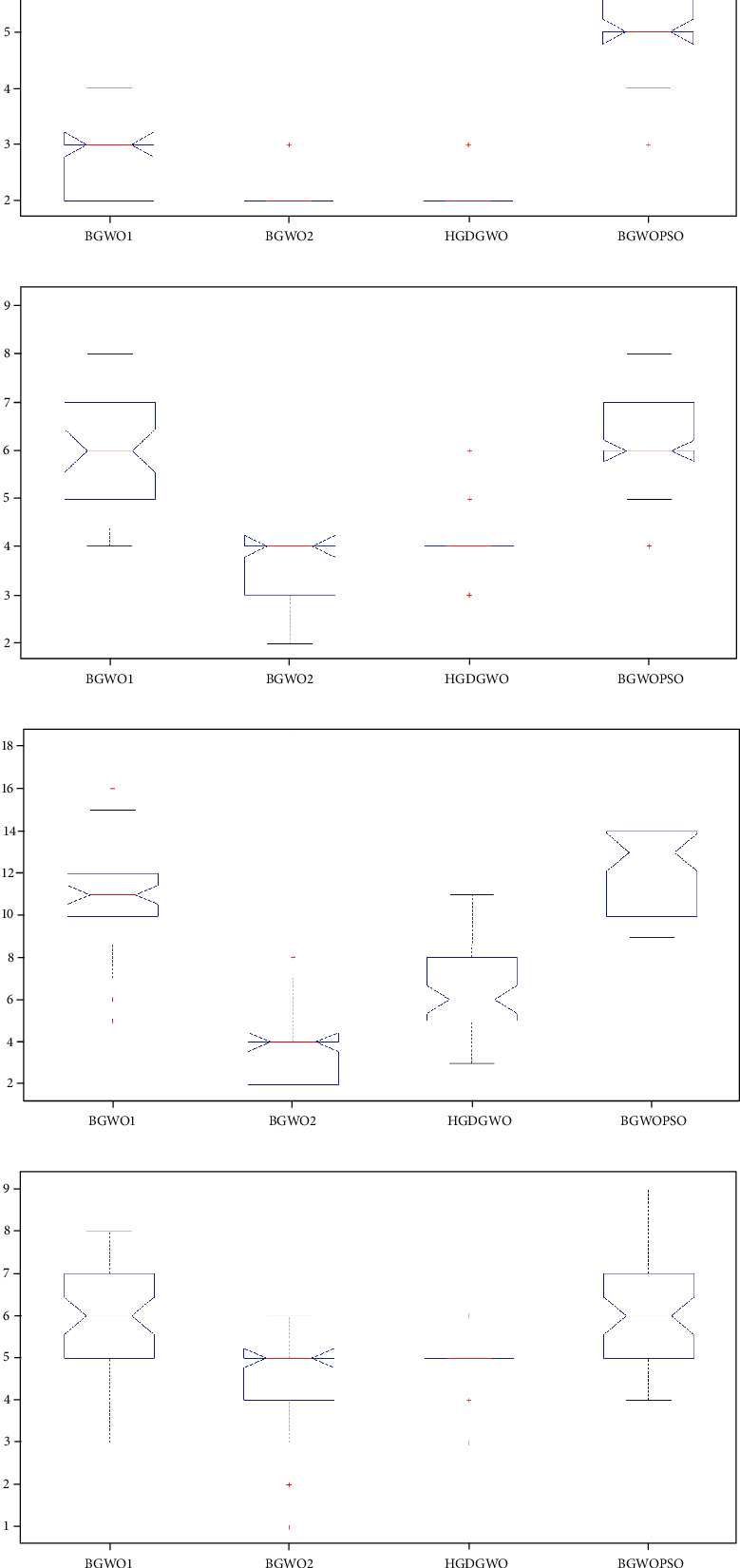
Box plots of the number of features.

As seen from Figures 26(a)–26(f), the proposed method is consistently among the lowest two median numbers of features for all the datasets in accordance with the objective of the algorithm, reducing feature subsets.

## 9. Conclusion and Future Work

The proposed hybrid gradient descent grey wolf optimizer encourages the worst-performing wolves to seek out the prey in the direction indicated by the leaders of the pack. The directional information is sourced from the partial derivative of the leading wolves.

The effectiveness of the proposed optimizer's first implementation was determined by evaluating its performance in feature optimization problems from the UCI datasets and compared to the BGWO2, outperforming it in 3 of the 4 datasets.

The second implementation outperforms BGWO implementations 1 and 2 in two datasets.

While the third implementation only completely outperforms the other algorithms (2 out of 6), the maximum accuracy tables show that the algorithm is capable of providing viable feature subsets even if the average may not be the best for different datasets, in 3 of 6 datasets. The margin between the best performing algorithm and the proposed algorithm is low with the maximum metrics always among the highest showing promise in the algorithm.

Improving the proposed algorithm would entail adding a memory module to the algorithm to reduce the computational load incurred in traversing the same points in the search space repeatedly.

Alternatively, a method may also be implemented to detect when the optimizer is stalling on a particular point on the search space, common in the local optimum. This may be modeled as a memory function that detects when the past *R* number of fitness values is constant, and if true, it increases randomization in the mutation function to allow the optimizer to tunnel out of the local optimum.

To reduce the overhead due to multiple partial derivative calculations, the features may be grouped into clusters with a single feature occurring in multiple clusters. The partial derivative is then calculated for all the bits changed in a cluster, and the partial derivative of each feature is calculated as the average of each cluster's partial derivative for which the feature occurs. This would reduce the number of calculations the algorithm calculates.

## Figures and Tables

**Figure 16:**
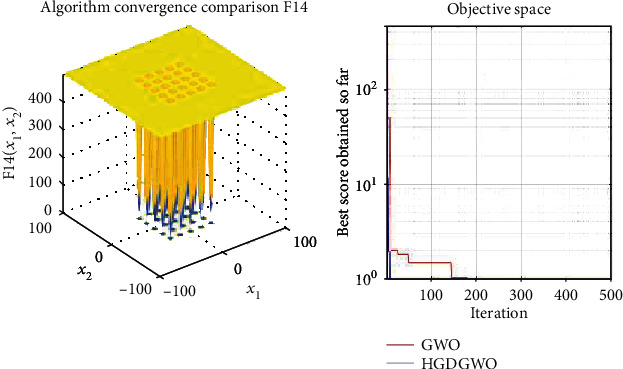
Convergence graph of fixed-dimension multimodal benchmark function (F14). GWO indicates grey wolf optimization; HGDGWO indicates hybrid gradient descent grey wolf optimizer.

**Figure 17:**
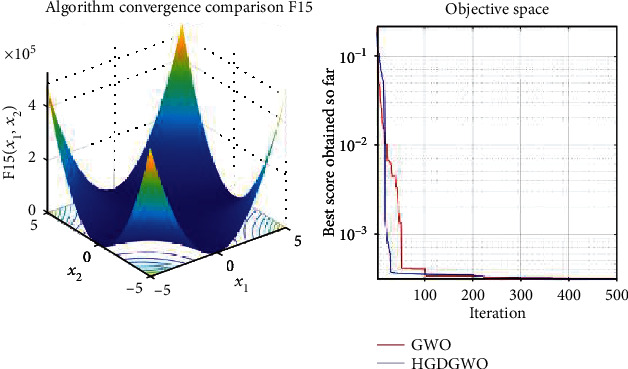
Convergence graph of fixed-dimension multimodal benchmark function (F15). GWO indicates grey wolf optimization; HGDGWO indicates hybrid gradient descent grey wolf optimizer.

**Figure 18:**
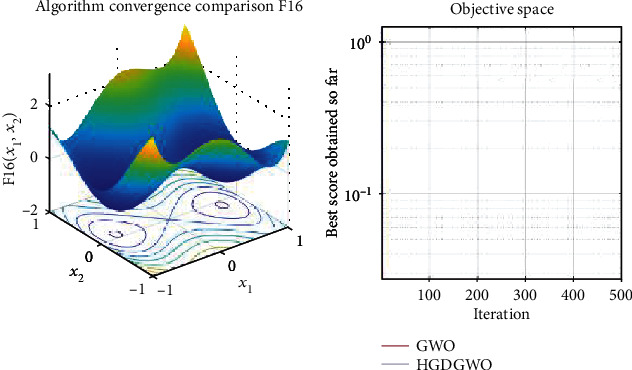
Convergence graph of fixed-dimension multimodal benchmark function (F16). GWO indicates grey wolf optimization; HGDGWO indicates hybrid gradient descent grey wolf optimizer.

**Figure 19:**
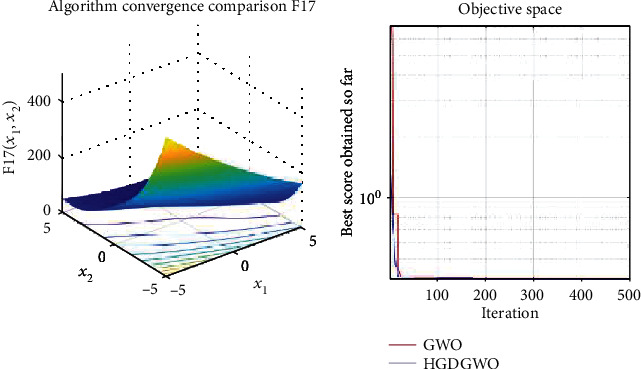
Convergence graph of fixed-dimension multimodal benchmark function (F17). GWO indicates grey wolf optimization; HGDGWO indicates hybrid gradient descent grey wolf optimizer.

**Figure 20:**
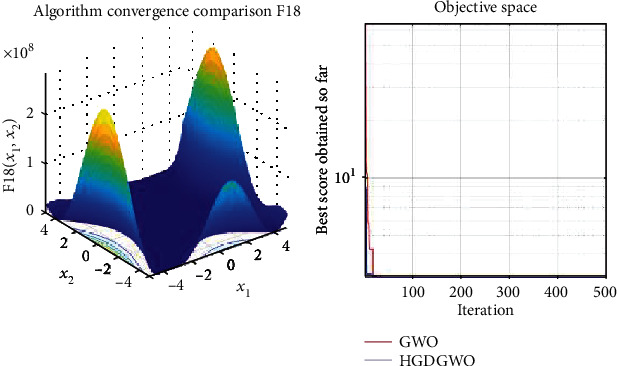
Convergence graph of fixed-dimension multimodal benchmark function (F18). GWO indicates grey wolf optimization; HGDGWO indicates hybrid gradient descent grey wolf optimizer.

**Figure 21:**
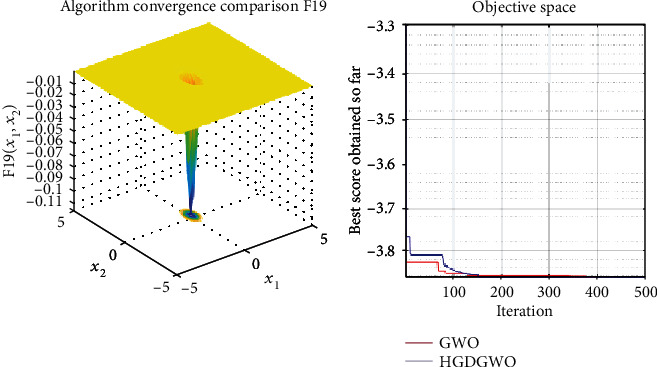
Convergence graph of fixed-dimension multimodal benchmark function (F19). GWO indicates grey wolf optimization; HGDGWO indicates hybrid gradient descent grey wolf optimizer.

**Figure 22:**
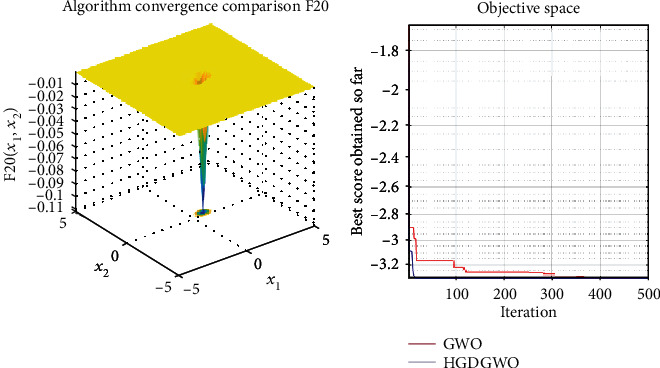
Convergence graph of fixed-dimension multimodal benchmark function (F20). GWO indicates grey wolf optimization; HGDGWO indicates hybrid gradient descent grey wolf optimizer.

**Figure 23:**
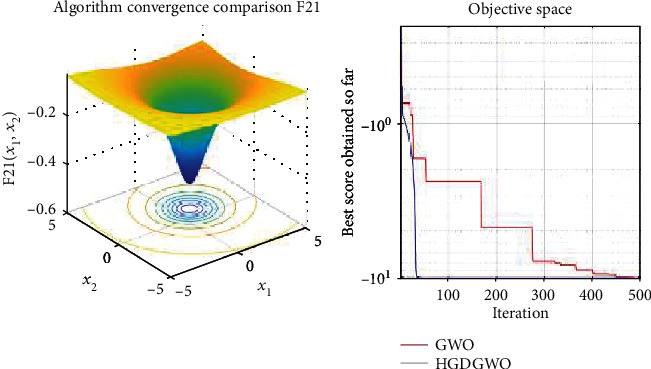
Convergence graph of fixed-dimension multimodal benchmark function (F21). GWO indicates grey wolf optimization; HGDGWO indicates hybrid gradient descent grey wolf optimizer.

**Figure 24:**
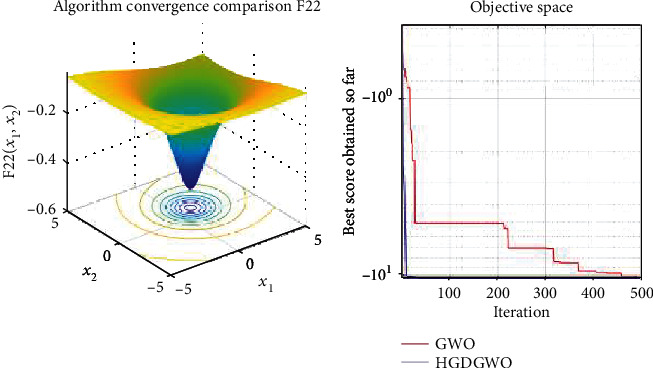
Convergence graph of fixed-dimension multimodal benchmark function (F22). GWO indicates grey wolf optimization; HGDGWO indicates hybrid gradient descent grey wolf optimizer.

**Figure 25:**
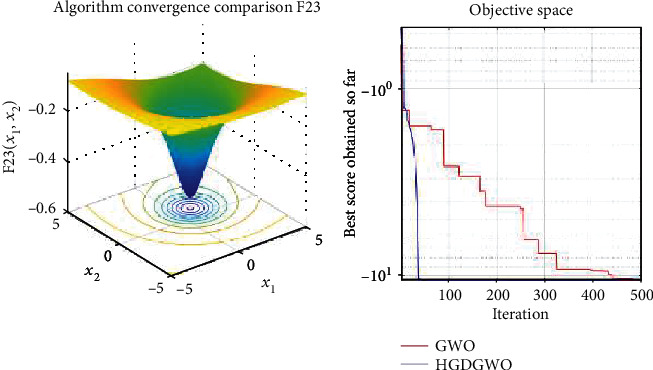
Convergence graph of fixed-dimension multimodal benchmark function (F23). GWO indicates grey wolf optimization; HGDGWO indicates hybrid gradient descent grey wolf optimizer.

**Pseudocode 1:**
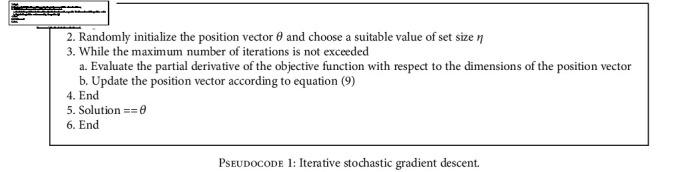
Iterative stochastic gradient descent.

**Pseudocode 2:**
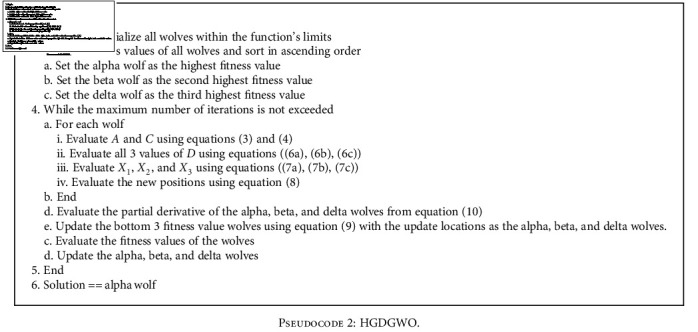
HGDGWO.

**Pseudocode 3:**
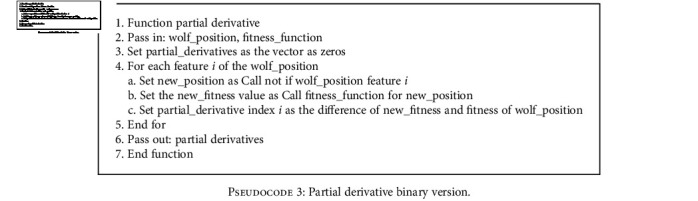
Partial derivative binary version.

**Pseudocode 4:**
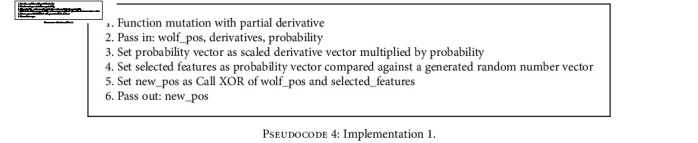
Implementation 1.

**Pseudocode 5:**
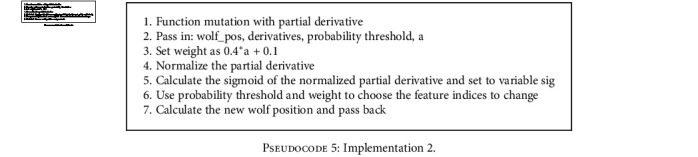
Implementation 2.

**Pseudocode 6:**
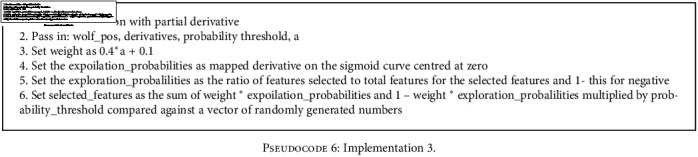
Implementation 3.

**Table 1:** Datasets for evaluating binary HGDGWO.

	No. of instances	No. of features
Breast Cancer Wisconsin (Diagnostic)	569	30
Breast Cancer Wisconsin (Original)	699	9
SPECT Heart	267	22
Statlog (Heart)	270	13
Heart Disease (Coronary Artery Disease)	303	14
Lymphography	148	18

**Table 2:** Comparison of HGDGWO vs. GWO on the standard deviation, average solution value, and minimum solution value.

	GWO			HGDGWO		
	Std	Avg	Min	Std	Avg	Min
F1	3.61676*E*-15	1.5*E*-15	1.05*E*-18	**2**.**552352E**-**16**	**1**.**12338E**-**16**	**3**.**42886E**-**19**
F2	5.10353*E*-10	6.14*E*-10	6.83*E*-11	**8**.**22765E**-**11**	**1**.**07201E**-**10**	**9**.**51197E**-**12**
F3	**1**.**970580354**	**0**.**440954**	**7**.**05E**-**05**	9.267454202	2.524266498	0.000739587
F4	**0**.**001694846**	**0**.**00097**	**2**.**45E**-**05**	0.627079638	0.194208881	9.11492*E*-05
F5	0.715412247	27.97616	26.07362	**0**.**182727572**	**24**.**70065652**	**24**.**2318205**
F6	0.549531169	2.059684	0.499325	**4**.**27267E**-**05**	**0**.**00015762**	**6**.**73711E**-**05**
F7	**0**.**002960497**	**0**.**00557**	**0**.**000473**	0.003179504	0.005692948	0.000678783
F8	864.6819996	-**5568**.**98**	-7969.82	**754**.**9053776**	-5331.436892	-**8200**.**684424**
F9	8.504768935	7.471358	**1**.**14E**-**13**	**4**.**948766233**	**5**.**290958807**	2.27374*E*-13
F10	4.48942*E*-09	6.1*E*-09	4.29*E*-10	**1**.**18025E**-**09**	**1**.**40909E**-**09**	**7**.**62208E**-**11**
F11	**0**.**015974785**	**0**.**009848**	1.11*E*-16	0.018615668	0.015057363	**0**
F12	**0**.**117619036**	0.164508	**0**.**011946**	0.161051964	**0**.**082169433**	0.016214596
F13	**0**.**291250797**	**1**.**478276**	**0**.**638533**	0.841310501	2.012431478	0.777870006
F14	**4**.**510397435**	**6**.**512168**	**0**.**998004**	4.84147902	7.537556634	0.998003838
F15	**0**.**009037547**	**0**.**005277**	0.000308	0.009730784	0.006187126	**0**.**000307503**
F16	1.22631*E*-07	-1.03163	-1.03163	**1**.**13088E**-**07**	-**1**.**031628364**	-**1**.**031628453**
F17	0.000315241	0.397931	**0**.**397887**	**1**.**68393E**-**05**	**0**.**397898059**	0.397887363
F18	20.41179783	8.508348	**3**	**13**.**87682254**	**5**.**667577926**	**3**.**000000005**
F19	0.127939439	-3.85542	-**3**.**86278**	**0**.**00129102**	-**3**.**86190632**	-3.862781552
F20	0.122039151	-3.24693	-3.32199	**0**.**060913623**	-**3**.**282130038**	-**3**.**321995138**
F21	**2**.**772143142**	-**8**.**63898**	-10.1531	2.959951738	-8.160509127	-**10**.**15318311**
F22	**1**.**692839418**	-**9**.**95648**	-10.4028	2.514675596	-9.309264282	-**10**.**40292471**
F23	**2**.**047149492**	-**9**.**94358**	-10.5363	2.972229183	-9.067428331	-**10**.**53639376**

**Table 3:** Comparison of HGDGWO vs. GWO on runtime in seconds.

	Timing in seconds	
	GWO	HGDGWO
F1	0.070489	0.282311792
F2	0.077059	0.365259492
F3	0.202226	2.946722992
F4	0.072127	0.392969492
F5	0.097035	0.375596492
F6	0.060202	0.224753292
F7	0.083923	0.723214392
F8	0.065475	0.359746292
F9	0.060371	0.267976592
F10	0.063073	0.323171992
F11	0.069045	0.410546392
F12	0.13222	1.664266292
F13	0.179679	2.283567892
F14	0.36951	0.539720892
F15	0.050027	0.093108092
F16	0.057001	0.116811892
F17	0.073093	0.115498092
F18	0.065085	0.080321992
F19	0.056733	0.106442192
F20	0.057225	0.145887392
F21	0.080076	0.202839092
F22	0.092606	0.244222492
F23	0.111739	0.314955192

**Table 4:** Results for HGDGWO (binary version) implementation 1.

		Accuracy	*F*-measure	Fitness values	No. of features	Precision	Sensitivity
Breast Cancer Wisconsin (Original)	JBGWO2	0.9401	0.9538	**0**.**0615**	**2**.**16**	0.9641	0.9448
	HGDGWO	**0**.**9442**	**0**.**9571**	0.064	2.28	**0**.**9642**	**0**.**9514**

Statlog (Heart)	JBGWO2	0.7889	0.8098	**0**.**1688**	**3**.**62**	0.8078	0.824
	HGDGWO	**0**.**7904**	**0**.**8125**	0.1805	3.98	**0**.**8109**	**0**.**828**

SPECT Heart	JBGWO2	**0**.**7161**	**0**.**7707**	**0**.**2304**	**3**.**94**	**0**.**7322**	**0**.**8213**
	HGDGWO	0.7017	0.7608	0.2532	6.38	0.7196	0.8112

Breast Cancer Wisconsin (Diagnostic)	JBGWO2	0.9361	0.9495	**0**.**0552**	**2**.**12**	0.9401	0.9602
	HGDGWO	**0**.**9596**	**0**.**9681**	0.0745	7.66	**0**.**9618**	**0**.**9753**

**Table 5:** Results of HGDGWO2, BGWO1, and BGWO2 on the Breast Cancer Wisconsin (Diagnostic) dataset.

	Breast Cancer Wisconsin (Diagnostic)								
	Average			Maximum			Minimum		
	BGWO1	BGWO2	HGDGWO	BGWO1	BGWO2	HGDGWO	BGWO1	BGWO2	HGDGWO
Accuracy	**0**.**962068**	0.938528	0.960664	**1**	**1**	**1**	**0**.**912281**	0.877193	**0**.**912281**
*F*-measure	**0**.**969959**	0.951626	0.968818	**1**	**1**	**1**	**0**.**929577**	0.901408	0.927536
Fitness values	0.088866	**0**.**054589**	0.07379	0.111523	**0**.**069948**	0.084128	0.068672	**0**.**043581**	0.065521
Number of features	11.76	**2**.**12**	7.68	17	**4**	11	7	**2**	5
Precision	**0**.**964718**	0.93962	0.964245	**1**	**1**	**1**	0.916667	0.888889	0.897436
Sensitivity	0.975952	0.964746	**0**.**974286**	**1**	**1**	**1**	0.914286	0.914286	0.888889

**Table 6:** Results of HGDGWO2, BGWO1, and BGWO2 on the Breast Cancer Wisconsin (Original) dataset.

	Breast Cancer Wisconsin (Original)								
	Average			Maximum			Minimum		
	BGWO1	BGWO2	HGDGWO	BGWO1	BGWO2	HGDGWO	BGWO1	BGWO2	HGDGWO
Accuracy	0.944807	0.94366	**0**.**948803**	0.985714	0.985714	**1**	**0**.**885714**	0.871429	**0**.**885714**
*F*-measure	0.957946	0.956807	**0**.**960627**	0.989011	0.989011	**1**	**0**.**911111**	0.907216	**0**.**911111**
Fitness values	0.065241	**0**.**061432**	0.063559	0.077354	**0**.**068009**	0.070794	**0**.**05227**	0.055132	0.055132
Number of features	2.64	**2**.**14**	2.28	4	**3**	**3**	**2**	**2**	**2**
Precision	0.95898	0.964994	**0**.**970211**	**1**	**1**	**1**	0.9	0.862745	0.901961
Sensitivity	**0**.**958135**	0.949845	0.952473	**1**	**1**	**1**	0.891304	0.891304	0.869565

**Table 7:** Results of HGDGWO2, BGWO1, and BGWO2 on the Statlog (Heart) dataset.

	Statlog (Heart)								
	Average			Maximum			Minimum		
	BGWO1	BGWO2	HGDGWO	BGWO1	BGWO2	HGDGWO	BGWO1	BGWO2	HGDGWO
Accuracy	0.777778	0.781481	**0**.**797778**	0.925926	**0**.**962963**	**0**.**962963**	**0**.**62963**	0.555556	0.555556
*F*-measure	0.805833	0.79969	**0**.**820237**	0.928571	**0**.**967742**	**0**.**967742**	**0**.**6875**	0.6	0.6
Fitness values	0.20767	**0**.**168917**	0.177561	0.287179	0.204558	**0**.**197721**	**0**.**149288**	**0**.**149288**	0.156695
Number of features	5.34	**3**.**74**	4.18	9	**5**	6	**3**	**3**	**3**
Precision	0.790494	0.817161	**0**.**820225**	**1**	**1**	**1**	0.608696	**0**.**6**	**0**.**6**
Sensitivity	**0**.**833333**	0.794667	**0**.**833333**	**1**	**1**	**1**	**0**.**6**	**0**.**6**	**0**.**6**

**Table 8:** Results of HGDGWO2, BGWO1, and BGWO2 on the SPECT Heart dataset.

	SPECT Heart								
	Average			Maximum			Minimum		
	BGWO1	BGWO2	HGDGWO	BGWO1	BGWO2	HGDGWO	BGWO1	BGWO2	HGDGWO
Accuracy	0.708091	**0**.**710598**	0.694758	0.814815	0.851852	**0**.**851852**	**0**.**555556**	0.518519	0.518519
*F*-measure	0.766021	**0**.**767059**	0.755479	0.857143	0.882353	**0**.**888889**	0.592593	**0**.**606061**	**0**.**606061**
Fitness values	0.258743	**0**.**229926**	0.254114	0.2825	**0**.**256591**	0.279545	0.226023	**0**.**209432**	0.228977
Number of features	11.06	**3**.**88**	7.06	14	**9**	12	5	**2**	**2**
Precision	0.724358	**0**.**727099**	0.71618	0.833333	0.833333	**0**.**875**	0.6	0.588235	0.588235
Sensitivity	**0**.**820417**	0.819583	0.806917	1	1	1	0.533333	0.625	0.625

**Table 9:** Average, maximum, and minimum metrics achieved for HGDGWO3, BGWOPSO, BGWO1, and BGWO2 on the Breast Cancer Wisconsin (Diagnostic) dataset.

	Breast Cancer Wisconsin (Diagnostic)											
	Average				Maximum				Minimum			
	BGWO1	BGWO2	BGWOPSO	HGDGWO	BGWO1	BGWO2	BGWOPSO	HGDGWO	BGWO1	BGWO2	BGWOPSO	HGDGWO
Accuracy	0.962	0.9375	**0**.**9684**	0.9533	**1**	0.9825	**1**	**1**	**0**.**9123**	0.8421	0.8947	0.8772
*F*-measure	0.9699	0.9511	**0**.**9751**	0.9631	**1**	0.9863	**1**	**1**	**0**.**9315**	0.8831	0.9167	0.9041
Fitness values	0.0911	**0**.**0573**	0.0658	0.07	0.1084	**0**.**0766**	0.0986	0.087	0.0771	0.0471	**0**.**0314**	0.0555
Number of features	11.72	**2**.**14**	11.98	5.96	16	**4**	18	9	9	**2**	**7**	3
Precision	0.9636	0.9369	**0**.**97**	0.9568	**1**	**1**	**1**	**1**	0.8974	0.8095	**0**.**9**	0.85
Sensitivity	0.977	0.9676	**0**.**9809**	0.9704	**1**	**1**	**1**	**1**	0.9143	0.8857	**0**.**9167**	0.8889

**Table 10:** Average, maximum, and minimum metrics achieved for HGDGWO3, BGWOPSO, BGWO1, and BGWO2 on the Breast Cancer Wisconsin (Original) dataset.

	Breast Cancer Wisconsin (Original)											
	Average				Maximum				Minimum			
	BGWO1	BGWO2	BGWOPSO	HGDGWO	BGWO1	BGWO2	BGWOPSO	HGDGWO	BGWO1	BGWO2	BGWOPSO	HGDGWO
Accuracy	0.945	0.9399	**0**.**9602**	0.9379	0.9857	0.9857	**1**	**1**	0.8286	0.8286	**0**.**913**	0.8286
*F*-measure	0.9574	0.9541	**0**.**9692**	0.9522	0.989	0.989	**1**	**1**	0.8636	0.8636	**0**.**9302**	0.8636
Fitness values	0.0654	0.0616	**0**.**0362**	0.0639	0.0806	0.068	**0**.**0538**	0.068	0.0551	0.0551	**0**.**0233**	0.058
Number of features	2.62	2.18	5.46	**2**.**14**	4	**3**	8	**3**	**2**	**2**	3	2
Precision	0.9671	0.9553	**0**.**9785**	0.9556	**1**	**1**	**1**	**1**	**0**.**9**	0.8824	**0**.**9**	**0**.**9**
Sensitivity	0.949	0.9542	**0**.**9611**	0.9499	**1**	**1**	**1**	**1**	0.8261	0.8261	**0**.**8889**	0.8261

**Table 11:** Average, maximum, and minimum metrics achieved for HGDGWO3, BGWOPSO, BGWO1, and BGWO2 on the Statlog (Heart) dataset.

	Statlog (Heart)											
	Average				Maximum				Minimum			
	BGWO1	BGWO2	BGWOPSO	HGDGWO	BGWO1	BGWO2	BGWOPSO	HGDGWO	BGWO1	BGWO2	BGWOPSO	HGDGWO
Accuracy	0.7815	0.7948	0.8	**0**.**8067**	0.9259	**0**.**963**	**0**.**963**	0.9259	0.6296	0.5556	**0**.**7037**	0.5556
*F*-measure	0.8043	0.8195	0.8218	**0**.**8327**	0.9333	**0**.**9677**	**0**.**9677**	0.9375	0.64	0.5714	**0**.**7407**	0.6
Fitness values	0.2068	**0**.**169**	0.1942	0.1765	0.3017	0.2598	0.2909	**0**.**1974**	0.1567	0.153	**0**.**1271**	0.1567
Number of features	6.02	**3**.**64**	6.24	3.9	8	**4**	9	6	4	**2**	4	3
Precision	0.8019	0.8023	**0**.**8213**	0.811	**1**	0.9375	**1**	0.9333	0.6667	0.6	**0**.**6842**	0.6
Sensitivity	0.8133	0.8413	0.8347	**0**.**8613**	**1**	**1**	**1**	**1**	0.5333	0.5333	**0**.**6667**	0.6

**Table 12:** Average, maximum, and minimum metrics achieved for HGDGWO3, BGWOPSO, BGWO1, and BGWO2 on the SPECT Heart dataset.

	SPECT Heart											
	Average				Maximum				Minimum			
	BGWO1	BGWO2	BGWOPSO	HGDGWO	BGWO1	BGWO2	BGWOPSO	HGDGWO	BGWO1	BGWO2	BGWOPSO	HGDGWO
Accuracy	0.6918	**0**.**7211**	0.6979	0.7066	**0**.**8889**	**0**.**8889**	**0**.**8889**	**0**.**8889**	**0**.**5185**	0.5	0.4231	0.5
*F*-measure	0.7524	**0**.**7775**	0.7605	0.7642	**0**.**9143**	**0**.**9143**	**0**.**9143**	0.9091	**0**.**6061**	0.6	0.5455	**0**.**6061**
Fitness values	0.2579	**0**.**2292**	0.2593	0.2483	**0**.**2916**	0.2515	0.3685	0.2801	0.2247	0.2086	**0**.**1943**	0.2244
Number of features	10.98	**3**.**7**	12.6	6.28	16	**8**	18	11	5	**2**	9	3
Precision	0.7181	**0**.**7336**	0.7154	0.7277	**0**.**9333**	0.8421	0.9333	0.8824	**0**.**5714**	0.5556	0.5	0.5556
Sensitivity	0.8006	**0**.**831**	0.8197	0.8119	**1**	**1**	**1**	0.9375	0.5625	0.6	0.6	**0**.**625**

**Table 13:** Average, maximum, and minimum metrics achieved for HGDGWO3, BGWOPSO, BGWO1, and BGWO2 on the Heart Disease (Coronary Artery Disease) dataset.

	Heart Disease (Coronary Artery Disease)											
	Average				Maximum				Minimum			
	BGWO1	BGWO2	BGWOPSO	HGDGWO	BGWO1	BGWO2	BGWOPSO	HGDGWO	BGWO1	BGWO2	BGWOPSO	HGDGWO
Accuracy	0.7909	0.7706	0.7838	**0**.**812**	**0**.**9667**	**0**.**9667**	**0**.**9667**	0.9355	0.5667	0.5161	0.6	**0**.**6129**
*F*-measure	0.7701	0.7386	0.7551	**0**.**7901**	0.963	**0**.**963**	**0**.**9655**	0.9333	0.5455	0.4444	0.5	**0**.**5833**
Fitness values	0.217	0.1869	**0**.**1909**	0.193	0.3077	0.2525	**0**.**2066**	0.3724	0.1741	0.1604	0.1703	**0**.**1195**
Number of features	6.12	**4**.**42**	4.88	6	8	**6**	**6**	**9**	3	**1**	3	4
Precision	0.7758	0.7706	0.7802	**0**.**8122**	**1**	**1**	**1**	**1**	0.5263	0.4615	**0**.**5714**	0.5625
Sensitivity	0.7786	0.7271	0.7457	**0**.**7875**	**1**	**1**	**1**	**1**	0.4286	0.4286	0.4286	**0**.**5**

**Table 14:** Average, maximum, and minimum metrics achieved for HGDGWO3, BGWOPSO, BGWO1, and BGWO2 on the Lymphography dataset.

	Lymphography											
	Average				Maximum				Minimum			
	BGWO1	BGWO2	BGWOPSO	HGDGWO	BGWO1	BGWO2	BGWOPSO	HGDGWO	BGWO1	BGWO2	BGWOPSO	HGDGWO
Accuracy	**0**.**5861**	0.5299	0.5165	0.5513	**0**.**6889**	0.5581	0.5833	0.6098	0.4878	**0**.**4889**	0.4545	0.4222
*F*-measure	0.6672	0.5808	0.5861	**0**.**6324**	**0**.**7667**	0.6275	0.6296	0.7143	**0**.**5714**	0.549	0.52	0.5
Fitness values	0.4751	0.4499	0.4759	**0**.**1832**	0.5365	0.5077	0.533	**0**.**2862**	0.4149	0.3926	0.417	**0**.**0763**
Number of features	8.9	**4**.**8**	6.2	9.7	10	**8**	9	13	8	**3**	4	6
Precision	**0**.**7017**	0.696	0.6628	0.6776	0.8824	**0**.**875**	0.875	0.8824	0.5	0.5	0.4815	0.**5185**
Sensitivity	**0**.**6775**	0.5536	0.5653	0.6444	0.85	0.7273	0.7143	**0**.**8571**	**0**.**4545**	0.4118	0.4375	0.3824

**Table 15:** Highest accuracy feature subset comparison of HGDGWO3, BGWOPSO, BGWO1, and BGWO2 on the Breast Cancer Wisconsin (Diagnostic) dataset.

Breast Cancer Wisconsin (Diagnostic)						
	Accuracy	*F*-measure	Fitness values	No. of features	Precision	Sensitivity
BGWO1	**1**	**1**	0.1033	12	**1**	**1**
BGWO2	0.9825	0.9863	**0**.**0541**	**2**	0.973	**1**
HGDGWO	**1**	**1**	0.069	7	**1**	**1**
BGWOPSO	**1**	**1**	0.0662	13	**1**	**1**

**Table 16:** Highest accuracy feature subset comparison of HGDGWO3, BGWOPSO, BGWO1, and BGWO2 on the Breast Cancer Wisconsin (Original) dataset.

Breast Cancer Wisconsin (Original)						
	Accuracy	*F*-measure	Fitness values	No. of features	Precision	Sensitivity
BGWO1	0.9857	0.989	0.0705	3	**1**	0.9783
BGWO2	0.9857	0.989	0.0651	2	**1**	0.9783
HGDGWO	**1**	**1**	0.068	**2**	**1**	**1**
BGWOPSO	**1**	**1**	**0**.**0475**	**6**	**1**	**1**

**Table 17:** Highest accuracy feature subset comparison of HGDGWO3, BGWOPSO, BGWO1, and BGWO2 on the Statlog (Heart) dataset.

Statlog (Heart)						
	Accuracy	*F*-measure	Fitness values	No. of features	Precision	Sensitivity
BGWO1	0.9259	0.9333	0.2017	6	0.9333	0.9333
BGWO2	**0**.**963**	**0**.**9677**	**0**.**1934**	3	**0**.**9375**	**1**
HGDGWO	0.9259	0.9333	0.1974	4	0.9333	0.9333
BGWOPSO	**0**.**963**	**0**.**9677**	0.2141	4	**0**.**9375**	**1**

**Table 18:** Highest accuracy feature subset comparison of HGDGWO3, BGWOPSO, BGWO1, and BGWO2 on the SPECT Heart dataset.

SPECT Heart						
	Accuracy	*F*-measure	Fitness values	No. of features	Precision	Sensitivity
BGWO1	**0**.**8889**	0.9032	0.2878	13	**0**.**9333**	0.875
BGWO2	**0**.**8889**	**0**.**9143**	**0**.**2491**	**2**	0.8421	**1**
HGDGWO	**0**.**8889**	0.9091	0.2801	8	0.8824	0.9375
BGWOPSO	**0**.**8889**	0.9032	0.2704	14	**0**.**9333**	0.875

**Table 19:** Highest accuracy feature subset comparison of HGDGWO3, BGWOPSO, BGWO1, and BGWO2 on the Heart Disease (Coronary Artery Disease) dataset.

Heart Disease (Coronary Artery Disease)						
	Accuracy	*F*-measure	Fitness values	No. of features	Precision	Sensitivity
BGWO1	**0**.**9667**	0.963	**0**.**189**	**4**	**1**	0.9286
BGWO2	**0**.**9667**	0.963	**0**.**189**	**4**	**1**	0.9286
HGDGWO	**0**.**9667**	**0**.**9655**	0.2033	5	0.9333	**1**
BGWOPSO	0.9355	0.9333	0.1517	8	0.875	**1**

**Table 20:** Highest accuracy feature subset comparison of HGDGWO3, BGWOPSO, BGWO1, and BGWO2 on the Lymphography dataset.

Lymphography						
	Accuracy	*F*-measure	Fitness values	No. of features	Precision	Sensitivity
BGWO1	**0**.**6889**	**0**.**7667**	0.5365	9	**0**.**8519**	0.697
BGWO2	0.5581	0.6275	0.3926	5	0.5517	**0**.**7273**
HGDGWO	0.5833	0.6296	0.5264	**5**	0.85	0.5
BGWOPSO	0.6098	0.7143	**0**.**2519**	8	0.8333	0.625

**Table 21:** *P* value test results for the number of features.

	Comparative *P* value			General *P* value
	BGWO1	BGWO2	BGWOPSO	
Breast Cancer Wisconsin (Diagnostic)	4.08*E*-40	1.88*E*-30	4.06*E*-25	5.71*E*-81
Breast Cancer Wisconsin (Original)	1.74*E*-06	0.58983	3.90*E*-36	2.53*E*-69
SPECT Heart	5.66*E*-19	2.11*E*-10	4.63*E*-24	1.00*E*-57
Statlog (Heart)	4.09*E*-21	0.0297	6.08*E*-19	4.08*E*-40
Heart Disease (Coronary Artery Disease)	0.00807	0.00864	0.4239528	1.11*E*-05
Lymphograpy	1.35*E*-07	0.0192	1.79*E*-07	1.89*E*-15

## Data Availability

The data used to support the findings of this study are available from the corresponding author upon request.
